# Comparative evaluation of ASCVD, SCORE-2, and HEARTS risk scores in Colombian adults at risk of type 2 diabetes: stratification, concordance, and associated factors

**DOI:** 10.3389/fcvm.2025.1734611

**Published:** 2026-02-04

**Authors:** Luis A. Anillo Arrieta, Yenifer Diaz Montes, Juan Jose Espitia De La Hoz, Tania Acosta-Vergara, Rafael Tuesca Molina, Victor Florez-Garcia, Jorge Acosta-Reyes, J. E. Rod, Pablo Aschner, Noël C. Barengo

**Affiliations:** 1 Division of Health Sciences, Department of Public Health, Universidad del Norte, Barranquilla, Colombia; 2 Division of Basic Sciences, Department of Mathematics and Statistics, Universidad del Norte, Barranquilla, Colombia; 3 School of Basic Sciences, Technology, and Engineering, Universidad Nacional Abierta y a Distancia–UNAD, Barranquilla, Colombia; 4 Faculty of Nursing Sciences, Universidad Cooperativa de Colombia, Santa Marta, Colombia; 5 Department of Preventive Medicine and Public Health, School of Medicine, Universidad Autónoma de Madrid, Madrid, Spain; 6 Division of Health Sciences, Department of Medicine, Universidad del Norte, Barranquilla, Colombia; 7 ScienceFlows Research Group, Universidad de Valencia, Valencia, España; 8 Division of Epidemiology and Biostatistics, School of Public Health, University of Illinois Chicago, Chicago, IL, United States; 9 Colombian Diabetes Association, Bogotá, Colombia; 10 Universidad Javeriana, Bogotá, Colombia; 11 Hospital Universitario San Ignacio, Bogotá, Colombia; 12 Department of Medical Education, Herbert Wertheim College of Medicine, Florida International University, Miami, FL, United States; 13 Escuela Superior de Medicina, Universidad Nacional de Mar del Plata, Mar del Plata, Argentina

**Keywords:** cardiovascular diseases, cardiovascular risk, diabetes mellitus type 2, risk assessment, risk factors

## Abstract

**Background:**

The Atherosclerotic Cardiovascular Disease Risk Estimator (ASCVD), Systematic Coronary Risk Evaluation (SCORE-2), and HEARTS risk scales are commonly used to estimate cardiovascular risk (CVR); however, their applicability may vary depending on the population context.

**Objective:**

This study aims to evaluate stratification, concordance, and associated clinical and social factors across three CVR scales in Colombian adults at risk of type 2 diabetes.

**Methods:**

A cross-sectional study was conducted among 868 adults. CVR was categorized as low, moderate, or high based on ASCVD, SCORE-2, and HEARTS criteria. Descriptive statistics were used for categorical variables, and comparisons were made using chi-square tests. Continuous variables were analyzed using the Kruskal–Wallis test. Concordance between scales was assessed using weighted Cohen's kappa (wκ), and ordinal logistic regression was applied to identify factors associated with CVR.

**Results:**

SCORE-2 classified 34.9% of participants as high cardiovascular risk, compared with 3.6% by ASCVD and 4.7% by HEARTS. Moderate agreement was observed between ASCVD and HEARTS (wκ = 0.49; *p* < 0.001). Living in Bogotá was associated with higher CVR according to ASCVD (OR 1.71; 95% CI: 1.28–2.30) and SCORE-2 (OR 1.74; 95% CI: 1.29–2.33). Higher education was protective in ASCVD and SCORE-2. Impaired glucose tolerance and type 2 diabetes were associated with increased CVR across all scales (*p* < 0.05).

**Conclusion:**

SCORE-2 classified a higher proportion of individuals as high cardiovascular risk, while ASCVD and HEARTS were more conservative. The low level of agreement between the scales highlights the need for local validation of CVR assessment tools.

## 1 Introduction

Cardiovascular disease (CVD) represents the leading cause of death globally, accounting for approximately 17.9 million deaths annually due to heart attacks and strokes. The burden of CVD varies across countries; in Latin America, it has been estimated that 35% of all deaths are attributable to CVD (1). Moreover, the prevalence of arterial hypertension (HTA) in Latin America affects one in four women and four in 10 men (2). In Colombia, CVD accounts for 28.7% of all deaths, and 22.8% of the population is affected by HTA (3). Therefore, in patients without established CVD, early identification of cardiovascular risk is essential, and cardiovascular risk (CVR) estimation represents the most important protective measure in primary prevention.

Various scales have been developed to estimate CVR, including the Atherosclerotic Cardiovascular Disease Risk Estimator (ASCVD) and the Systematic Coronary Risk Evaluation (SCORE-2), both of which are widely used in American and European populations (4–6). However, their application in regions with different sociodemographic, ethnic, genetic, environmental, and healthcare profiles requires prior validation to ensure clinical accuracy. The World Health Organization (WHO) has developed and validated global CVR prediction models across 21 regions (7). In the Americas, the Pan American Health Organization (PAHO) promotes the HEARTS initiative as a primary care-based strategy, which has been implemented in Colombia since 2015 and adopted by 22 countries, aiming for full deployment by 2025 (8, 9).

Despite these efforts, limitations remain, as tools like ASCVD and SCORE-2 were developed primarily in high-income countries with minimal representation from Latin American populations. In response, regional adaptations, such as HEARTS in the Americas, have been introduced (10–12). However, the coexistence of multiple cardiovascular risk assessment tools introduces complexity, as patients may be classified differently depending on the scale used, leading to inconsistent treatment decisions (11). These tools also differ in included variables and outcome measures (6, 13, 14), and such heterogeneity complicates both the clinical application and the administrative and financial planning within healthcare systems.

In addition to classic risk factors, metabolic conditions like prediabetes increase the likelihood of progressing to type 2 diabetes (T2D), CVD, and cerebrovascular events (CVEs). According to the International Diabetes Federation (IDF), individuals with T2D have a 72% higher risk of myocardial infarction, a 52% higher risk of CVE, and an 84% higher risk of heart failure (1, 15). These data underscore the need for effective CVR assessment tools in this population. Although several CVR scales are available, their performance in Colombian adults at risk for T2D remains unclear. Addressing this gap could enhance risk detection and inform prevention strategies in Colombia, Latin America, and other resource-limited settings. Therefore, the present study aimed to evaluate stratification, concordance, and associated clinical and social factors of three CVR scales in Colombian adults at risk of type 2 diabetes.

## 2 Methods

### 2.1 Study design and population

This analytical cross-sectional study used data from the PREDICOL Project (Clinical Trials.gov identifier NCT03049839). The detailed methodology of the project has been described in previous publications (16–20).

**Figure 1:**
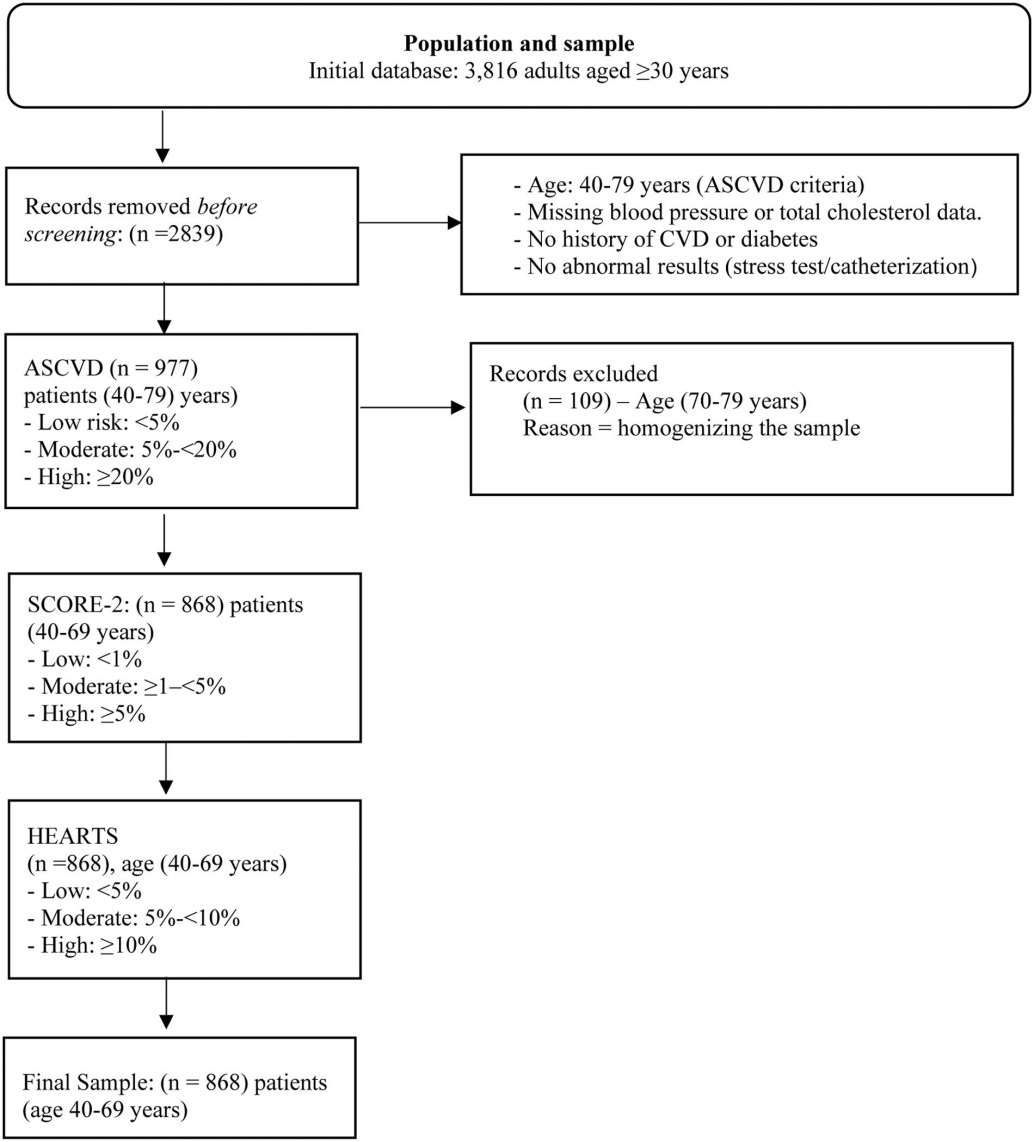
Flowchart describing the sample selection process. Risk categories are standardized as “low,” “moderate,” and “high” in the text, although the original thresholds of each tool are respected.

The initial study database included 3,816 adults aged over 30 years. For the cardiovascular risk analysis, inclusion criteria established by the ASCVD scale were applied, as this tool only considers individuals aged 40–79 years with complete blood pressure and total cholesterol data, no history of cardiovascular disease or diabetes, and no abnormal findings on stress testing or catheterization. Consequently, 2,839 records were excluded, yielding an intermediate sample of 977 participants eligible for the application of the ASCVD scale. Subsequently, 109 individuals aged 70–79 years were excluded because this age range is not contemplated by the SCORE-2 or HEARTS in the Americas models. Thus, the final sample comprised 868 participants aged 40–69 years, homogeneous in terms of age and clinical criteria, to whom all three cardiovascular risk scales (ASCVD, SCORE-2, and HEARTS) were uniformly applied (Figure 1).

### 2.2 Variables and measurements

The ASCVD, SCORE-2, and HEARTS assessment tools were selected based on their international endorsement, applicability in primary prevention, and prior use or validation in Latin American populations (6, 13, 14, 21). To enable cross-tool comparison, we additionally divided the participants into three cardiovascular risk categories (low, moderate, and high) according to internationally recognized thresholds used in global cardiovascular prevention frameworks.

#### 2.2.1 Atherosclerotic Cardiovascular Disease risk estimator

The ASCVD risk estimator, developed by the American College of Cardiology (ACC) and American Heart Association (AHA), assesses both10-year and lifetime risk of atherosclerotic cardiovascular disease in individuals without prior cardiovascular events. Based on the pooled cohort equations (PCEs), the tool has been validated in multicenter studies, such as the Multi-Ethnic Study of Atherosclerosis (MESA), supporting its applicability across diverse populations, including African Americans (13). It incorporates age, total cholesterol, HDL, systolic blood pressure (SBP), antihypertensive treatment, T2D, and smoking status. Outcomes include fatal and non-fatal myocardial infarction and cerebrovascular events. Applicable to individuals aged 40–79, the ASCVD risk estimator classifies risk into four categories: low (<5%), borderline (5% to <7.5%), intermediate (7.5% to <20%), and high (≥20%). For this study, these categories were reclassified into three groups—(1) low (<5%), (2) moderate (5% to <20%), and (3) high (≥20%)—to align with other scales and facilitate comparison.

#### 2.2.2 Systematic Coronary Risk Evaluation

SCORE-2 estimates the 10-year risk of fatal and non-fatal cardiovascular events, including myocardial infarction, stroke, and other atherosclerotic diseases. Unlike the original SCORE model, which focused solely on cardiovascular mortality, SCORE-2 broadens its scope and is intended for individuals aged 40–69 years, while its complementary version, SCORE-2-OP, is intended for those aged 70 years and older (6, 21). It includes age, sex, systolic blood pressure, total and HDL cholesterol, and smoking status and categorizes risk as low (<1%), moderate (≥1–<5%), high (≥5% up to 10%), and very high (>10%) (6, 21). Although SCORE-2 has been widely adopted in clinical guidelines, its predictive performance in non-European populations remains under evaluation. In this study, only the SCORE-2 version was used and was limited to individuals aged 40–69 to match the defined sample; for comparison across cardiovascular risk scales, SCORE-2 risk categories were recategorized into low (<1%), moderate (≥1 to <5%), and high (≥5%).

#### 2.2.3 Hearts in the Americas (HEARTS)

HEARTS is a WHO/PAHO tool for estimating 10-year CVR of myocardial infarction, CVEs, and cardiovascular death, while also supporting hypertension management in primary care settings. Validated in six subregions and implemented in 21 countries, it incorporates age (40–75), sex, history of CVD, diabetes, chronic kidney disease, smoking status, systolic blood pressure (90–200 mmHg), BMI, and total cholesterol (140–300 mg/dL) (14). Cardiovascular risk is classified into five color-coded categories to facilitate visual interpretation: low (<5%), green; moderate (5% to <10%), yellow; high (10% to <20%), orange; very high (20% to <30%), red; and extreme (≥30%), dark red,. The HEARTS app incorporates country-specific epidemiological data and is freely available in Spanish, English, and Portuguese for both iOS and Android. Designed for clinical use, especially in primary care, it also includes protocols, guidelines, and educational tools for public use (14). In this study, HEARTS risk categories were regrouped as follows: (1) low (<5%), (2) moderate (5% to <10%), and (3) high (≥10%).

#### 2.2.4 Other clinical and social determinants measures of CVR

Sociodemographic variables included age, sex, city of residence (Barranquilla or Bogotá D.C.), marital status, educational level, and family history of diabetes. Clinical variables included BMI (kg/m^2^), classified as normal (18.5–24.9), overweight (25.0–29.9), or obese (≥30.0), and waist circumference, categorized as normal or increased using sex-specific cutoffs (<94/≥94 cm for men; <80/≥80 cm for women) (22). Physical activity was defined as ≥30 min of activity per day or ≥4 h of activity per week. Antihypertensive medication use was recorded as a binary variable. Glycemic status was assessed using an oral glucose tolerance test (OGTT) in participants with a FINDRISC score of 12 or higher. Classification followed WHO criteria: type 2 diabetes (FPG ≥126 mg/dL or 2-hPG ≥200 mg/dL), impaired fasting glucose (FPG 110–125 mg/dL), impaired glucose tolerance (2-hPG ≥140 mg/dL), and normoglycemia (FPG <110 mg/dL and 2-hPG <140 mg/dL). Prediabetes was defined as the presence of IFG, IGT, or both (23).

### 2.3 Statistical analyses

Categorical variables were summarized as frequencies and percentages, with group differences assessed using the chi-square test. Continuous variables were tested for normality (Kolmogorov–Smirnov); as distributions were non-normal, results are reported as medians and percentiles; group comparisons were performed using the Kruskal–Wallis test. Agreement between the ASCVD, SCORE-2, and HEARTS scales was assessed using weighted Cohen's kappa (w*κ*), with pairwise comparisons and stratification by glycemic status; 95% confidence intervals (CI) were reported. Multicollinearity was assessed using correlation matrices and variance inflation factors (VIF <5). To avoid redundancy, variables already included in the CVR scores (BMI, family history, fasting glucose, blood pressure, antihypertensive use) were excluded. Ordinal logistic regression models, both unadjusted and adjusted, were fitted for each risk scale and included city, education, marital status, physical activity, fruit intake, waist circumference, LDL, and OGTT classification. ORs with 95% CI were reported. Model adequacy was confirmed using the parallel lines test (*p* > 0.05). Pseudo-*R*² and AIC were used to evaluate model performance. Analyses were performed using STATA software, version 17.0.

### 2.4 Ethics statement

The study was approved by the Ethics Committee of the Universidad del Norte (Document No. 141, issued 28 April 2016). All participants voluntarily signed informed consent and retained the right to withdraw from the study at any time. The research was conducted in accordance with Good Clinical Practice (GCP) standards and the principles established in the Declaration of Helsinki (24).

## 3 Results

### 3.1 Sample characteristics

The study included 868 participants, of whom 77.2% were women, with a mean age of 54.9 years (SD ± 7.91). A family history of T2D in first- or second-degree relatives was reported by 50.5% of participants. SCORE-2 classified the highest proportion of individuals as high risk (303 participants; 34.91%), compared to ASCVD (31 participants; 3.57%) and HEARTS (41 participants; 4.72%). Table 1 shows significant differences in cardiovascular risk distribution with respect to age, sex, city, education, and glycemic status (*p* < 0.001). Higher cardiovascular risk was more common among participants aged ≥60 years, men, residents of Bogotá, individuals with lower educational attainment, and those with T2D. Age-stratified patterns were particularly pronounced across the three scales: ASCVD showed a shift toward moderate and high risk among older adults, SCORE-2 classified the majority of participants aged ≥60 years as high risk due to its strong weighting of age, and HEARTS similarly concentrated high-risk classifications among older individuals. In addition, family history of diabetes, smoking, and antihypertensive use were associated with a higher probability of being classified in the moderate or high-risk categories, whereas BMI showed no significant association with cardiovascular risk.

**Table 1:** Participants' characteristics by cardiovascular risk scale.

Variables	Total	ASCVD				SCORE-2				HEARTS			
		Low	Moderate	High	*p*-Value	Low	Moderate	High	*P*-Value	Low	Moderate	High	*P*-Value
	868 (100%)	489 (56.34)	348 (40.09)	31 (3.57)		45 (5.18)	520 (59.91)	303 (34.91)		675 (77.76)	152 (17.51)	41 (4.72)	
Age (years)													
<60	591 (68.1)	477 (91.4)	143 (41.1)	1 (3.3)	**0** **.** **000**	45 (100.0)	463 (89.0)	83 (27.4)	**0** **.** **000**	537 (79.6)	51 (33.5)	3 (7.3)	**0** **.** **000**
≥60	277 (31.9)	42 (8.6)	205 (58.9)	30 (96.7)		0 (0.0)	57 (11.0)	220 (72.6)		138 (20.4)	101 (66.5)	38 (92.7)	
Gender													
Male	198 (22.8)	46 (9.4)	125 (35.9)	27 (87.1)	**0** **.** **000**	0 (0.0)	71 (13.6)	127 (41.9)	**0** **.** **000**	97 (14.3)	72 (47.3)	29 (70.7)	**0** **.** **000**
Female	670 (77.1)	443 (90.5)	223 (64.0)	4 (12.9)		45 (100.0)	449 (86.3)	176 (58.0)		578 (85.6)	80 (52.6)	12 (29.2)	
City													
Barranquilla	492 (56.6)	306 (62.5)	172 (49.4)	14 (45.1)	**0** **.** **000**	33 (73.3)	316 (60.7)	143 (47.1)	**0** **.** **000**	362 (53.6)	107 (70.3)	23 (56.1)	**0** **.** **000**
Bogotá DC	376 (43.3)	183 (37.4)	176 (50.5)	17 (54.8)		12 (26.6)	204 (39.2)	160 (52.8)		313 (46.3)	45 (29.6)	18 (43.9)	
Marital status													
Single	144 (16.5)	78 (15.9)	63 (18.1)	3 (9.6)	0.337	3 (6.6)	85 (16.3)	56 (18.4)	0.207	113 (16.7)	27 (17.7)	4 (9.7)	0.347
Married	555 (63.9)	323 (66.0)	213 (61.2)	19 (61.2)		35 (77.7)	336 (64.6)	184 (60.7)		425 (62.9)	103 (67.7)	27 (65.8)	
Divorced-widowed	169 (19.4)	19.47 (18.0)	72 (20.6)	9 (29.0)		7 (15.5)	63 (20.7)	99 (19.0)		137 (20.3)	22 (14.4)	10 (24.3)	
Education level													
No schooling	27 (3.1)	13 (2.6)	9 (2.5)	5 (16.1)	**0** **.** **000**	0 (0.0)	14 (2.6)	13 (4.2)	**0** **.** **000**	21 (3.1)	3 (1.9)	3 (7.3)	**0** **.** **027**
Primary	413 (47.5)	190 (38.8)	208 (59.7)	15 (48.3)		8 (17.7)	227 (43.6)	178 (58.7)		307 (45.4)	83 (54.6)	23 (56.1)	
Secondary	331 (38.1)	213 (43.5)	109 (31.3)	9 (29.0)		25 (55.5)	212 (40.7)	94 (31.0)		261 (38.6)	55 (36.1)	15 (36.5)	
Higher education	97 (11.1)	73 (14.9)	22 (6.3)	2 (6.4)		12 (26.6)	67 (12.8)	18 (5.9)		86 (12.7)	11 (7.2)	0 (0.0)	
Physical activity													
No	784 (90.3)	449 (91.8)	307 (88.2)	28 (90.3)	0.221	39 (86.6)	480 (92.3)	265 (87.4)	**0** **.** **053**	618 (91.5)	134 (88.1)	32 (78.0)	**0** **.** **010**
Yes	84 (9.6)	40 (8.1)	41 (11.7)	3 (9.6)		6 (13.3)	40 (7.6)	38 (12.5)		57 (8.4)	18 (11.8)	9 (21.9)	
Consumption of fruits and vegetables													
No	746 (85.9)	419 (85.6)	297 (85.3)	30 (96.7)	0.208	38 (84.4)	448 (86.1)	260 (85.8)	0.947	577 (85.4)	135 (88.8)	34 (82.9)	0.480
Yes	122 (14.0)	70 (14.3)	51 (14.6)	1 (3.2)		7 (15.5)	72 (13.8)	43 (14.1)		98 (14.5)	17 (11.1)	7 (17.0)	
Hypertension treatment													
No	583 (67.1)	385 (78.7)	185 (53.1)	13 (41.9)	**0** **.** **000**	45 (100.0)	373 (71.7)	165 (54.4)	**0** **.** **000**	482 (71.4)	81 (53.2)	20 (48.7)	**0** **.** **000**
Yes	285 (32.8)	104 (21.2)	163 (46.8)	18 (58.0)		0 (0.0)	147 (28.2)	138 (45.5)		193 (28.5)	71 (46.7)	21 (51.2)	
BMI (kg/m^2^)													
Normal	124 (14.2)	75 (15.3)	44 (12.6)	5 (16.1)	0.838	7 (15.5)	75 (14.4)	42 (13.8)	0.873	96 (14.2)	22 (14.4)	6 (14.6)	0.897
Overweight	370 (42.6)	206 (42.1)	152 (43.6)	12 (38.7)		16 (35.5)	226 (43.4)	128 (42.2)		283 (41.9)	70 (46.0)	17 (41.4)	
Obese	374 (43.0)	208 (42.5)	152 (43.6)	14 (45.1)		22 (48.8)	219 (42.1)	133 (43.8)		296 (43.8)	60 (39.4)	18 (43.9)	
Family history of diabetes													
No	263 (30.3)	115 (23.5)	133 (38.2)	15 (48.3)	**0** **.** **000**	8 (17.7)	135 (25.9)	120 (39.6)	**0** **.** **000**	185 (27.4)	59 (38.8)	19 (46.3)	**0** **.** **008**
Yes, 1st y 2nd degree	438 (50.4)	275 (56.2)	151 (43.3)	12 (38.7)		29 (64.4)	279 (53.6)	130 (42.9)		352 (52.1)	71 (46.7)	15 (36.5)	
Yes 3rd y 4th degree	167 (19.2)	99 (20.2)	64 (18.3)	4 (12.9)		8 (17.7)	106 (20.3)	53 (17.4)		138 (20.4)	22 (14.4)	7 (17.0)	
Smoking													
No	795 (91.5)	476 (97.3)	295 (84.7)	24 (77.4)	**0** **.** **000**	45 (199.0)	497 (95.5)	253 (83.5)	**0** **.** **000**	617 (91.4)	143 (94.0)	35 (85.3)	0.190
Yes	73 (8.4)	13 (2.6)	53 (15.2)	7 (22.5)		0 (0.0)	23 (4.4)	50 (16.5)		58 (8.5)	9 (5.9)	6 (14.6)	
Oral glucose tolerance test (OGTT)													
NGT	26 (3.0)	17 (3.4)	9 (2.5)	0 (0.0)	**0** **.** **000**	1 (2.2)	18 (3.4)	7 (2.3)	0.109	19 (2.8)	7 (4.6)	00 (0.0)	**0** **.** **000**
IFG	112(12.9)	55(11.2)	51(14.6)	6(19.3)		1(2.2)	61(11.7)	50(16.5)		72(10.6)	31(20.3)	9(21.9)	
IGT	665(76.6)	399(81.6)	249(71.5)	17(54.8)		40(88.8)	404(77.6)	221(72.9)		540(80.0)	100(65.7)	25(60.9)	
T2D	65(7.4)	18(3.6)	39(11.2)	8(25.8)		3(6.6)	37(7.1)	25(8.2)		44(6.5)	14(9.2)	7(17.0)	

Homogeneity test (*χ*²); *n* (%). OGTT, oral glucose tolerance test; NGT, patients with normoglycemia; prediabetes, patients with impaired fasting glucose (IFG), IGT, impaired glucose tolerance; T2D, type 2 diabetes mellitus; BMI, body mass index. Cardiovascular risk (CVR) was assessed using three scoring systems: SCORE-2, which estimates 10-year risk of cardiovascular incidence and mortality; ASCVD, which estimates 10-year risk of atherosclerotic cardiovascular disease incidence; and HEARTS in the Americas, which estimates 10-year risk of acute myocardial infarction (AMI), stroke, and cardiovascular mortality.

Bold values indicate statistically significant differences (*p* < 0.05).

### 3.2 Scale evaluation and agreement

**Figure 2:**
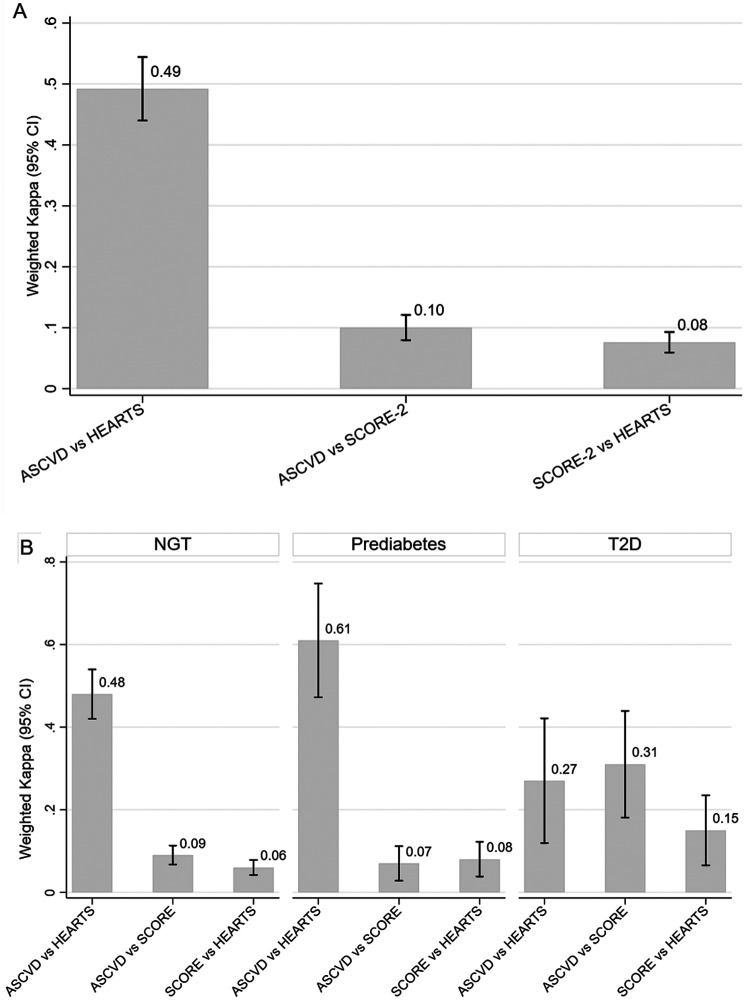
Concordance of cardiovascular risk scores across glycemic states demonstrating a comparative analysis of ASCVD, SCORE-2, and HEARTS. SCORE-2 classified the highest proportion of participants as high risk 303 (34.91%), compared to ASCVD 31 (3.57%) and HEARTS 41 (4.72%). (**A**) Weighted Cohen's kappa (wκ) values with 95% confidence intervals (CIs) for agreement across three cardiovascular risk classification scales: ASCVD, SCORE-2, and HEARTS. Weighted Cohen's kappa coefficients were 0.10 (95% CI: 0.08–0.13) for ASCVD vs. SCORE-2, 0.49 (95% CI: 0.44–0.54) for ASCVD vs. HEARTS, and 0.08 (95% CI: 0.06–0.11) for SCORE-2 vs. HEARTS. (**B**) Stratified agreement by glycemic status: normal glucose tolerance (NGT), prediabetes, and type 2 diabetes mellitus (T2D). Bars represent weighted kappa values, and vertical lines indicate 95% CIs. Values closer to 1 indicate stronger agreement.

The distribution of cardiovascular risk according to the ASCVD, SCORE-2, and HEARTS scales is presented in Figure 2, showing differences in risk categorization trends across the tools. Figure 2A presents agreement between scales. The highest concordance was observed between ASCVD and HEARTS (wκ = 0.49; 95% CI: 0.44–0.54). Lower agreement was observed between ASCVD and SCORE-2 (wκ = 0.10) and between SCORE-2 and HEARTS (wκ = 0.08), with both *p*-values being <0.001. Figure 2B shows stratified analyses. ASCVD and HEARTS showed the highest agreement among participants with prediabetes (wκ = 0.61; 95% CI: 0.47–0.74), followed by those with normoglycemia (wκ = 0.48; 95% CI: 0.42–0.54) and T2D (wκ = 0.27; 95% CI: 0.13–0.41). SCORE-2 comparisons showed consistently low concordance across all glycemic groups (wκ = 0.06–0.15). A complementary table summarizing the original classification criteria of the three risk scales is included in the Supplementary Material.

### 3.3 Association between clinical characteristics, social determinants, and scales

The results of the bivariate relationship between T2D diabetes risk variables and CVR scales are summarized in Table 2. Two-hour postprandial glucose levels were significantly higher in individuals classified as high ASCVD risk (130 mg/dL, IQR 97–175). A similar pattern was observed for the HEARTS in the Americas (WHO/PAHO), in which 2-h postprandial glucose levels increased from 96 mg/dL (IQR 82–109) in the low-risk group to 109 mg/dL (IQR 91–135) in the high-risk group (*p* = 0.001). However, postprandial glucose levels did not show statistically significant differences across SCORE-2 risk categories (*p* = 0.361). Waist circumference, fasting glucose, and LDL levels did not differ significantly across risk categories for any of the three cardiovascular risk scales (*p* > 0.05). Table 3 presents the ordinal logistic regression models for ASCVD, SCORE-2, and HEARTS (WHO/PAHO) cardiovascular risk classifications. SCORE-2 showed the best predictive performance (pseudo-*R*^2^ = 0.078; *χ*^2^ = 109.399, *p* < 0.001), followed by ASCVD (pseudo-*R*^2^ = 0.060). HEARTS showed the lowest explanatory capacity (pseudo-*R*^2^ = 0.045) but exhibited the greatest parsimony, with lower AIC and BIC values. All models satisfied the proportional odds assumption (parallel lines test, *p* > 0.05).

**Table 2:** Distribution of clinical characteristics by cardiovascular risk stratification in adults at risk for T2D.

Variables	ASCVD					SCORE-2				HEARTS			
	Total	Low	Moderate	High	*p*-Value	Low	Moderate	High	*P*-Value	Low	Moderate	High median	*P*-Value
	Median	Median	Median	Median		Median	Median	Median	Median	Median	Median	Median	
	(Q1–Q3)	(Q1–Q3)	(Q1–Q3)	(Q1–Q3)		Median	Median	Median	Median	(Q1–Q3)	(Q1–Q3)	(Q1–Q3)	
OGTT	105 (87–129)	100 (84–122)	109 (92–135)	130 (97–175)	0.001	101 (86–123)	112.5 (95–142)	122 (97–167)	0.361	96 (82–109)	102.5 (86–126)	109 (91–135)	0.001**
Wais circumference	99 (93–105)	97 (92–103)	100 (95–107)	100 (95–110)	0.353	98 (92–104)	101 (94–108)	101 (96–107)	0.345	97 (92–101)	98 (92–104)	100 (94–107)	0.353
Basal glycemia	93 (85–101)	92 (85–100)	94 (87–102)	101 (91–114)	0.100	93 (85–101)	95 (85–101)	97 (89–109)	0.416	85 (81–93)	93.5 (85–101)	94 (87–101)	0.100
LDL	114.3 (95–141)	114.2 (95–141)	114.7 (92–141)	115 (101–147)	0.389	114.2 (95–141)	114.7 (92–141)	115 (101–147)	0.180	104.6 (82–121)	113.7 (93–139)	120.1 (98–148)	0.389

LDL, low-density lipoprotein. *P*-value: ‡Kruskal–Wallis test. No marks: not significant.

* *P* < 0.05.

** *P* < 0.001; IQR, interquartile range: (Q1–Q3).

**Table 3:** Factors associated with cardiovascular risk scales in the study participants.

	ASCVD^b^				SCORE-2^c^				HEARTS^d^			
	OR^a^ unadjusted (95% IC)	*p*-Value	OR^a^ adjusted (95% CI)	*P*-Value	OR^a^ unadjusted (95% IC)	*p*-Value	OR^a^ adjusted (95% CI)	*p*-Value	OR^a^ unadjusted (95% IC)	*p*-Value	OR^a^ adjusted (95% CI)	*p*-Value
City												
Barranquilla	**Ref.**		**Ref.**		**Ref.**		**Ref.**		**Ref.**		**Ref.**	
Bogota, DC	1.72 (1.32–2.25)	0.001***	1.71 (1.28–2.30)	0.001***	1.84 (1.40–2.42)	0.001***	1.74 (1.29–2.33)	0.001***	0.57 (0.41–0.81)	0.001**	0.57 (0.40–0.82)	0.002***
Education level|												
No schooling	**Ref.**		**Ref.**		**Ref.**		**Ref.**		**Ref.**		**Ref.**	
Elementary school	0.77 (0.34–1.74)	0.532	0.90 (0.39–2.10)	0.247	0.79 (0.37–1.70)	0.558	0.82 (0.37–1.79)	0.622	1.10 (0.43–2.83)	0.828	1.05 (0.40–2.75)	0.918
Junior high school	0.37 (0.16–0.85)	0.02**	0.49 (0.21–1.16)	0.118	0.38 (0.17–0.83)	0.016**	0.43 (0.19–0.95)	0.038**	0.86 (0.33–2.22)	0.761	0.79 (0.29–2.10)	0.64
Superior	0.22 (0.08–0.56)	0.001***	0.28 (0.10–0.73)	0.009***	0.21 (0.08–0.50)	0.001***	0.23 (0.09–0.56)	0.001***	0.40 (0.13–1.21)	0.105	0.33 (0.10–1.05)	0.062
Marital status												
Single	Ref.		Ref.		Ref.		Ref.		Ref.		Ref.	
Divorced-widower	1.14 (0.74–1.77)	0.54	1.11 (0.70–1.76)	0.652	0.89 (0.57–1.39)	0.626	0.83 (0.53–1.32)	0.448	0.89 (0.51–1.54)	0.686	0.85 (0.48–1.50)	0.593
Free union-married	0.87 (0.61–1.26)	0.48	0.96 (0.65–1.41)	0.852	0.72 (0.50–1.03)	0.080	0.74 (0.50–1.08)	0.127	1.13 (0.73–1.76)	0.567	1.01 (0.64–1.60)	0.953
Physical activity												
No	Ref.		Ref.		Ref.		Ref.		Ref.		Ref.	
Yes	1.43 (0.92–2.21)	0.110	1.69 (1.06–2.69)	0.026	1.47 (0.93–2.31)	0.094	1.72 (1.07–2.77)	0.024**	1.85 (1.14–3.01)	0.013**	2.21 (1.33–3.66)	0.002***
Daily fruit intake												
No	Ref.		Ref.		Ref.		Ref.		Ref.		Ref.	
Yes	0.90 (0.61–1.32)	0.605	0.90 (0.60–1.36)	0.645	0.99 (0.67–1.46)	0.992	1.09 (0.72–1.63)	0.678	0.85 (0.53–1.38)	0.525	0.77 (0.47–1.28)	0.327
Waist circunference												
No	Ref.		Ref.		Ref.		Ref.		Ref.		Ref.	
Yes	1.71 (1.07–2.74)	0.024**	1.57 (0.96–2.56)	0.072	1.46 (0.93–2.31)	0.001***	1.34 (0.84–2.15)	0.212	1.24 (0.71–2.16)	0.435	1.10 (0.62–1.94)	0.741
LDL												
Normal	Ref.		Ref.		Ref.		Ref.		Ref.		Ref.	
Altered	1.69 (1.28–2.22)	0.001***	1.60 (1.20–2.14)	0.001***	1.62 (1.23–2.14)	0.001***	1.47 (1.10–1.96)	0.009***	1.11 (0.80–1.54)	0.64	1.28 (0.91–1.81)	0.154
OGTT												
NTG	Ref.		Ref.		Ref.		Ref.		Ref.		Ref.	
IFG	0.77 (0.34–1.74)	0.532	0.77 (0.33–1.80)	0.56	0.83 (0.37–1.84)	0.653	0.82 (0.36–1.85)	0.648	1.47 (0.61–3.53)	0.196	1.80 (0.73–4.38)	0.196
IGT	1.59 (1.07–2.36)	0.021**	1.85 (1.21–2.81)	0.004***	1.73 (1.17–2.57)	0.006***	2.10 (1.38–3.19)	0.001***	2.38 (1.55–3.64)	0.001***	2.19 (1.40–3.42)	0.001***
T2D	4.19 (2.47–7.10)	0.001***	4.60 (2.69–7.87)	0.001***	1.26 (0.76–2.11)	0.36	1.26(0.74–2.14)	0.392	2.16(1.24–3.75)	0.001***	2.01(1.14–3.54)	0.015**

Altered waist circumference in women ≥80 cm and in men ≥94 cm.

a Ordinal logistic regression model, proportional odds, odds ratio (95% confidence interval). Ref., reference group.

b Pseudo-*R*^2^ 0.078, chi-square 109.399, prob > chi^2^ 0.000, Akaike criterion (AIC) 1,324.539, Bayesian criterion (BIC) 1,396.032. Test of parallel lines (likelihood ratio): *P*-value = 0.105.

c Pseudo-*R*^2^ 0.060, chi-square 86.754, prob > chi^2^ 0.001 Akaike criterion (AIC) 1,380.251, Bayesian criterion (BIC) 1,451.744. Test of parallel lines (likelihood ratio): *P*-value = 0.063.

d Pseudo- *R*^2^ 0.045, chi-square 50.669, prob > chi^2^ 0.000 Akaike criterion (AIC) 1,098.805, Bayesian criterion (BIC) 1,170.298. Test of parallel lines (likelihood ratio): *P*-value = 0.999.

* Statistically significant *p*-value < 0.05. OGTT, oral glucose tolerance test; NGT, patients with normoglycemia; prediabetes, patients with impaired fasting glucose (IFG); IGT, patients with impaired glucose tolerance; T2D, type 2 diabetes mellitus.

*** *p* < 0.01.

** *p* < 0.05.

#### 3.3.1 ASCVD

Residence in Bogotá was associated with a higher likelihood of classification into higher ASCVD categories compared to residence in Barranquilla (OR 1.71, 95% CI: 1.28–2.30). University education showed a protective effect vs. no formal education (OR 0.28, 95% CI: 0.10–0.73). Elevated LDL cholesterol increased the odds of a higher ASCVD category (OR 1.60, 95% CI: 1.20–2.14). Impaired glucose tolerance was also linked to greater risk (OR 1.85, 95% CI: 1.21–2.81), and type 2 diabetes was associated with a markedly increased risk (OR 4.60, 95% CI: 2.69–7.87). In contrast, marital status, daily fruit intake, and waist circumference did not show significant associations with ASCVD risk (*p* > 0.05).

#### 3.3.2 SCORE-2

Residence in Bogotá was associated with higher odds of classification into higher SCORE-2 risk categories compared to residence in Barranquilla (OR 1.74, 95% CI: 1.29–2.33). Both secondary (OR 0.43, 95% CI: 0.19–0.95) and university (OR 0.23, 95% CI: 0.09–0.56) education showed protective effects compared to no formal education. Elevated LDL cholesterol (OR 1.47, 95% CI: 1.10–1.96) and impaired glucose tolerance (OR 2.10, 95% CI: 1.38–3.19) were associated with a higher risk. Physical activity was also associated with greater odds of classification into high SCORE-2 risk categories (OR 1.72, 95% CI: 1.07–2.77). Marital status, daily fruit intake, waist circumference, and type 2 diabetes showed no significant associations (*p* > 0.05).

#### 3.3.3 Hearts in the Americas (WHO/PAHO)

Residence in Bogotá was associated with lower odds of classification into higher HEARTS cardiovascular risk categories compared to residence in Barranquilla (OR 0.57, 95% CI: 0.40–0.82). Impaired glucose tolerance (OR 2.19, 95% CI: 1.40–3.42) and type 2 diabetes (OR 2.01, 95% CI: 1.14–3.54) were associated with a higher cardiovascular risk. Physical activity was also associated with an increased odds of classification into higher HEARTS risk categories (OR 2.21, 95% CI: 1.33–3.66). Educational level, marital status, daily fruit intake, waist circumference, and altered LDL levels were not significantly associated (*p* > 0.05).

## 4 Discussion

This study compared the ASCVD, SCORE-2, and HEARTS cardiovascular risk scales in 868 Colombian adults and demonstrated differences in patient risk classification across tools. SCORE-2 identified the largest proportion of individuals as high risk, while HEARTS tended to classify fewer participants in higher risk categories. The low concordance between scales highlights the need for local validation, as differences in predicted outcomes, methods, and context limit their interchangeability. To facilitate comparison across tools, we applied a harmonized three-category risk classification (low, moderate, and high risk), a strategy commonly used in comparative cardiovascular research. Categorization of continuous risk scores is a widespread and accepted practice in epidemiology (25, 26), especially when it enhances interpretability and supports model comparison. This strategy also aligns with reporting guidelines that emphasize the importance of transparent justification for grouping decisions (27). Moreover, statistical m such as reclassification analyses rely on predefined categories to assess agreement and predictive performance (28). In a recent example, Luca et al. demonstrated that application of different cardiovascular guidelines to the same high-risk population yielded significant reclassification, reinforcing the value of standardized risk categories in comparative studies (29).

The OGTT emerged as the most sensitive metabolic marker across all cardiovascular risk scales. ASCVD showed stronger associations with waist circumference, LDL cholesterol, and T2D; HEARTS was more closely associated with IGT, physical activity, and residence in Barranquilla; and SCORE-2 demonstrated stronger associations with educational level. Notably, cardiovascular risk was substantially higher among adults aged ≥60 years across the three scales. SCORE-2 showed the greatest age dependency, classifying most older adults as high risk, whereas ASCVD and HEARTS also demonstrated clear upward shifts in risk categories among participants aged ≥60 years. Existing evidence indicates that socioeconomically disadvantaged populations experience higher rates of cardiovascular events and face structural barriers to timely prevention and treatment (30). In this context, CVD risk stratification is essential for guiding cost-effective interventions, particularly in resource-limited settings. The WHO scales offer versions with and without laboratory tests, enabling their application according to diagnostic availability (31). While simplified cardiovascular models are useful in low-income settings (32, 33), adding clinical biomarkers may improve prediction. However, the relative contribution of clinical, biochemical, and sociodemographic factors, especially in populations at high metabolic risk, remains unclear. This issue is particularly relevant for preventing CVD in individuals at high risk of T2D. Incorporating social determinants may further enhance predictive capacity and contextualization (34). The variability in cardiovascular risk classification is consistent with previous studies in Latin America. Bazo-Alvarez et al. found low concordance among six CVR scales applied to diverse, low-income Peruvian populations (35). In Colombia, similar discrepancies have been reported between models such as Framingham, SCORE-2, and AHA/ACC (36, 37). While the Framingham score tends to overestimate risk in individuals without a cardiovascular history (AUC = 0.65), the sex-adjusted PROCAM model showed superior performance (AUC = 0.74; expected/observed ratio = 1.07), indicating greater discrimination and calibration (38).

In addition, recent research recommends recalibrating international models to include sex- and context-specific factors, as they often overestimate risk in middle- and low-income countries (39). In Colombia, SCORE-2 has been shown to overestimate cardiovascular risk (fatal and non-fatal myocardial infarction, stroke, heart failure, and angina) by 22% in women and 42% in men (39). Given this overestimation and its lower concordance with the other scales, ASCVD may provide more accurate estimates, while HEARTS tends to classify the same individuals as low risk. This is relevant since the WHO recommends initiating pharmacological prevention based on risk stratification (40), and applying risk scales developed for high-income countries to low- and middle-income settings could lead to overprescription and inefficient resource use. SCORE-based models remain among the three most commonly used guidelines in Colombia (36). These findings underscore the limitations of current risk stratification tools for clinical practice and public health use, particularly in the absence of economic evaluations of their impact (36).

Overall, the present study findings suggest that SCORE-2 may overestimate cardiovascular risk if it is assumed that ASCVD and HEARTS perform a more accurate risk estimation in individuals at risk for T2D. Although the SCORE-2 scale demonstrated the greatest explanatory power (pseudo-*R*^2^ of 0.078), this does not guarantee better individual-level clinical discrimination. The HEARTS tool is useful in primary care due to its simplicity; however, it omits several critical variables, which may limits its predictive capacity (41). It also lacks factors such as family history, socioeconomic status, and inflammatory biomarkers, potentially limiting prediction in some subgroups. For ASCVD (pseudo-*R*^2^ = 0.060), unmeasured factors, including lifestyle or genetics, may influence classification. Agreement between cardiovascular risk scales varied according to glycemic status: in normoglycemic participants, the tendency of HEARTS to classify individuals as low CVR could avoid overtreatment; in those with prediabetes, ASCVD offered a balanced risk stratification; and in participants with T2D, agreement across scales was low; however, SCORE-2 and ASCVD demonstrated the highest relative agreement, suggesting that SCORE-2 may be preferable when overestimation is considered safer. An unexpected finding was the positive association between self-reported physical activity and higher SCORE-2 risk, likely due to subjective measurement, lack of duration/intensity data, and possible MET underestimation in older adults (42). Prior studies have shown that physical activity levels exceeding 26.5 MET-h/week reduce the risk of metabolic syndrome (43) and that achieving ≥1000 MET-min/week reduces mortality by 14% (44). However, physical activity patterns are also influenced by factors such as age, education, and geography (45), which were not fullyaccounted for in the present analysis.

In addition, the present study identified advanced age, male sex, low educational level, antihypertensive treatment, family history of diabetes, and impaired glucose tolerance as factors significantly associated with higher cardiovascular risk in at least one of the evaluated scales. Selvin et al. reported chronic hyperglycemia as an independent risk factor for CVD, even in the absence of a formal diabetes diagnosis (46), supporting its role in endothelial damage, chronic inflammation, and atherosclerosis progression (47, 48). A dose–response relationship was observed, with cardiovascular risk increasing from normoglycemia to prediabetes and undiagnosed T2D, consistent with results from the PURE study, which reported higher CVD mortality in diabetes patients from low- and middle-income countries (39). Absolute cardiovascular risk is greater when diabetes develops at younger ages, underscoring the value of markers such as glycated hemoglobin (49). From a pathophysiological perspective, altered glycemic metabolism contributes to CVD through endothelial dysfunction, oxidative stress, lipid profile changes, and chronic inflammation, promoting atherosclerosis and plaque instability (47, 48). Prediabetes and impaired glucose tolerance represent intermediate but clinically relevant stages along this risk continuum. Tests such as the OGTT and HbA1c should be prioritized for early detection in primary care, especially in contexts where delayed diagnosis is common (49). These findings highlight the importance of assessing cardiovascular risk assessment scales within specific subpopulations, such as individuals at risk for T2D.

Of particular relevance for primary health care strategies is the finding that clinical variables and social determinants such as waist circumference, LDL cholesterol, and T2D were more strongly associated with ASCVD, while IGT, physical activity, and residence in Barranquilla were more closely associated with HEARTS. These variables may serve as practical predictors for the application of risk assessment scales by untrained personnel or in resource-limited settings. Educational level was the only variable more strongly linked to SCORE-2, although only slightly more so than with ASCVD, raising the question of whether clinical and social variables could be used to index SCORE-2 results. Waist circumference may represent a useful anthropometric marker for monitoring cardiovascular risk (50), potentially reducing follow-up time in low-complexity patients, whereas the OGTT may be more valuable in complex cases, given its superior predictive capacity. Further studies are needed to identify which clinical variables are most effective for this role.

Among the main strengths of this study is its comparative approach of validated risk scales in a Latin American population at risk for T2D. The inclusion of sociodemographic, clinical, and metabolic variables provides a comprehensive assessment of cardiovascular risk. However, limitations include the cross-sectional design, which precludes causal inference, and self-reported physical activity without objective measures, potentially generating spurious associations, as suggested by studies questioning generic METs in older adults (42). Likewise, the use of harmonized risk categories to facilitate comparison across scales may have reduced some of the nuance inherent in the original thresholds. In addition, differences in prediction horizons and reference populations across models may introduce minor constraints when interpreting the results. The low pseudo-*R*^2^ values (ASCVD: 0.060; HEARTS: 0.045) indicate that unmeasured factors, such as genetics, diet, mental health, or environmental exposures, may influence cardiovascular risk. Despite these considerations, this study provides meaningful evidence on the comparative performance of cardiovascular risk stratification tools among individuals at risk for T2D. Our findings offer initial guidance on the limited agreement between the three scales in this specific subpopulation and highlight clinical variables that may help contextualize risk assessment and disease progression in primary health care settings.

## 5 Conclusions

Our findings suggest that the OGTT may be a better predictor of cardiovascular risk than LDL and waist circumference. Although overall agreement across the evaluated tools was low, ASCVD and HEARTS demonstrated moderate concordance compared with SCORE-2, which classified the largest proportion of participants as high risk. Different CVR scales may be more appropriate at distinct pathophysiological stages along the diabetes risk continuum. Further studies are needed to validate these results, adapt models to local contexts, and evaluate their performance in diverse populations. Improved measurement of physical activity using validated instruments and the inclusion of socioeconomic factors and inflammatory biomarkers could enhance cardiovascular risk stratification in Colombia.

## Data Availability

The raw data supporting the conclusions of this article will be made available by the authors, without undue reservation.

## References

[1] World Health Organization. Cardiovascular diseases (2025). Available online at: https://www.who.int/health-topics/cardiovascular-diseases (Accessed February 13, 2025).

[2] Campbell NRC Paccot Burnens M Whelton PK Angell SY Jaffe MG Cohn J 2021 World Health Organization guideline on pharmacological treatment of hypertension: policy implications for the region of the Americas. Rev Panam Salud Publica. (2022) 46:e54. 10.26633/RPSP.2022.54 35573116 PMC9097923

[3] Rivas SR Serna Tobón DC Gallego KYM Cardona MPT Spitia JDC Gutierrez PAM Impacto de la iniciativa HEARTS en una institución de salud de segundo nivel en Colombia. Rev Panam Salud Publica. (2022) 46:e152. 10.26633/RPSP.2022.152 36133427 PMC9484327

[4] Soudri R Dharmadhikari S Khandhedia C Mane A Mehta S. Assessment of atherosclerotic cardiovascular disease risk using atherosclerotic cardiovascular disease risk estimator plus in Indian patients—a real-world, retrospective study from electronic medical records. Indian Heart J. (2024) 76:S149–50. 10.1016/j.ihj.2024.11.302

[5] Conroy RM Pyörälä K Fitzgerald AP Sans S Menotti A De Backer G Estimation of ten-year risk of fatal cardiovascular disease in Europe: the SCORE project. Eur Heart J. (2003) 24(11):987–1003. 10.1016/S0195-668X(03)00114-3 12788299

[6] Graham IM Di Angelantonio E Visseren F De Bacquer D Ference BA Timmis A Systematic coronary risk evaluation (SCORE). J Am Coll Cardiol. (2021) 77(24):3046–57. 10.1016/j.jacc.2021.04.052 34140109 PMC8091419

[7] Kaptoge S Pennells L De Bacquer D Cooney MT Kavousi M Stevens G World Health Organization cardiovascular disease risk charts: revised models to estimate risk in 21 global regions. Lancet Glob Health. (2019) 7(10):e1332–45. 10.1016/S2214-109X(19)30318-3 31488387 PMC7025029

[8] Li J Liu F Yang X Cao J Chen S Chen J Validating world health organization cardiovascular disease risk charts and optimizing risk assessment in China. Lancet Reg Health West Pac. (2021) 8:100096. 10.1016/j.lanwpc.2021.100096 34327424 PMC8315380

[9] Flood D Edwards EW Giovannini D Ridley E Rosende A Herman WH HEARTS Como herramienta para integrar el manejo de la hipertensión y la diabetes en los entornos de atención primaria de salud. Rev Panam Salud Publica. (2022) 46:213. 10.26633/RPSP.2022.213 PMC9673610 36415785

[10] Abate KH Abebe Z Abil OZ Afshin A Ahmed MB Alahdab F Global, regional, and national incidence, prevalence, and years lived with disability for 354 diseases and injuries for 195 countries and territories, 1990–2017: a systematic analysis for the Global Burden of Disease Study 2017. Lancet. (2018) 392(10159):1789–858. 10.1016/S0140-6736(18)32279-7 30496104 PMC6227754

[11] Murray CJ Ezzati M Flaxman AD Lim S Lozano R Michaud C GBD 2010: design, definitions, and metrics. Lancet. (2012) 380(9859):2063–6. 10.1016/S0140-6736(12)61899-6 23245602

[12] Murray CJL Lopez AD. Measuring global health: motivation and evolution of the global burden of disease study. Lancet. (2017) 390(10100):1460–4. 10.1016/S0140-6736(17)32367-X 28919120

[13] Wong ND Budoff MJ Ferdinand K Graham IM Michos ED Reddy T Atherosclerotic cardiovascular disease risk assessment: an American society for preventive cardiology clinical practice statement. Am J Prev Cardiol. (2022) 10:100335. 10.1016/j.ajpc.2022.100335 35342890 PMC8943256

[14] Ordunez P Tajer C Gaziano T Rodriguez YA Rosende A Jaffe MG. The HEARTS app: a clinical tool for cardiovascular risk and hypertension management in primary health care. Rev Panam Salud Publica. (2022) 46:12. 10.26633/RPSP.2022.12 PMC8959249 35355690

[15] International Diabetes Federation. . IDF Diabetes Atlas. 11th ed Brussels: IDF (2025). Available online at: https://diabetesatlas.org/resources/idf-diabetes-atlas-2025/ (Accessed February 13, 2025).

[16] Acosta T Tuesca R Florez K Barengo NC Anillo L Flórez-García V Factors associated with low physical activity in two Latin American populations at risk of developing type 2 diabetes: an exploratory analysis. Front Public Health. (2021) 8:589484. 10.3389/fpubh.2020.589484 33520912 PMC7842278

[17] Anillo Arrieta LA Acosta Vergara T Tuesca R Rodríguez Acosta S Flórez Lozano KC Aschner P Health-related quality of life (HRQoL) in a population at risk of type 2 diabetes: a cross-sectional study in two Latin American cities. Health Qual Life Outcomes. (2021) 19(1):189. 10.1186/s12955-021-01894-7 34930297 PMC8686566

[18] Anillo Arrieta LA Flórez Lozano KC Tuesca Molina R Acosta Vergara T Rodríguez Acosta S Aschner P Glycemic status and health-related quality of life (HRQOL) in populations at risk of diabetes in two Latin American cities. Qual Life Res. (2023) 32(8):2361–73. 10.1007/s11136-023-03398-x 37010804 PMC10328894

[19] Montes YD Vergara TA Molina RT Guerrero GM Arrieta LAA Aschner P The association between sociodemographic characteristics, clinical indicators and body mass index in a population at risk of type 2 diabetes: a cross-sectional study in two Colombian cities. Prim Care Diabetes. (2024) 18(4):458–65. 10.1016/j.pcd.2024.06.001 38862312

[20] Montes YD Anillo Arrieta LA De La Hoz JJE Acosta-Vergara T Acosta-Reyes J Flórez Lozano KC Effectiveness of a community intervention program on healthy lifestyles (PREDICOL) among adults with prediabetes in two Latin American cities: a quasi-experimental study. Prim Care Diabetes. (2025) 19(3):277–87. 10.1016/j.pcd.2025.03.010 40158901

[21] Tomasik T Krzysztoń J Dubas-Jakóbczyk K Kijowska V Windak A. The systematic coronary risk evaluation (SCORE) for the prevention of cardiovascular diseases. Does evidence exist for its effectiveness? A systematic review. Acta Cardiol. (2017) 72(4):370–9. 10.1080/00015385.2017.1335052 28705107

[22] ⁠Ministry of Health and Social Protection of Colombia. Evaluate your weight (2025). Available online at: https://www.minsalud.gov.co/salud/Paginas/Evalue-su-peso.aspx (Accessed February 21, 2025).

[23] ⁠ Ministry of Health and Social Protection of Colombia. Clinical practice guideline for the diagnosis of type II diabetes. Available online at: https://www.minsalud.gov.co/sites/rid/Lists/BibliotecaDigital/RIDE/DE/CA/gpc-pacientes-diabetes-mellitus-tipo2-poblacion-mayor-18-anos.pdf (Accessed February 21, 2025).

[24] World Medical Association. WMA Declaration of Helsinki. Ethical principles for medical research. Available online at: https://www.wma.net/policies-post/wma-declaration-of-helsinki/ (Accessed February 21, 2025).

[25] Turner EL Dobson JE Pocock SJ. Categorisation of continuous risk factors in epidemiological publications: a survey of current practice. Epidemiol Perspect Innov. (2010) 7:9. 10.1186/1742-5573-7-9 20950423 PMC2972292

[26] Mabikwa OV Greenwood DC Baxter PD Fleming SJ. Assessing the reporting of categorised quantitative variables in observational epidemiological studies. BMC Health Serv Res. (2017) 17:201. 10.1186/s12913-017-2137-z 28288628 PMC5348776

[27] Pocock SJ Collier TJ Dandreo KJ de Stavola BL Goldman MB Kalish LA Issues in the reporting of epidemiological studies: a survey of recent practice. Br Med J. (2004) 329(7471):883. 10.1136/bmj.38250.571088.55 15469946 PMC523109

[28] Cook NR Paynter NP. Performance of reclassification statistics in comparing risk prediction models. Biometrical J. (2011) 53(2):237–58. 10.1002/bimj.201000078 PMC3395053 21294152

[29] Luca SA Bungau RM Lazar S Potre O Timar B. To what extent does cardiovascular risk classification of patients with type 2 diabetes differ between European guidelines from 2023, 2021, and 2019? A cross-sectional study. Medicina (Kaunas). (2024) 60(2):334. 10.3390/medicina60020334 38399621 PMC10890196

[30] Hastings K Marquina C Morton J Abushanab D Berkovic D Talic S Projected new-onset cardiovascular disease by socioeconomic group in Australia. Pharmacoeconomics. (2022) 40(4):449–60. 10.1007/s40273-021-01127-1 35037191 PMC8761535

[31] Rezaei F Seif M Gandomkar A Fattahi MR Malekzadeh F Sepanlou SG Comparison of laboratory-based and non-laboratory-based WHO cardiovascular disease risk charts: a population-based study. J Transl Med. (2022) 20(1):336. 10.1186/s12967-022-03336-4 35296342 PMC8925162

[32] Momeni M Danaei M Ebrahimi S. Estimating the frequency of risk factors and the 10-year risk of developing cardiovascular diseases in middle-aged population in Kerman, Iran. Shiraz E Med J. (2019) 21(1):e90551. 10.5812/semj.90551

[33] Rezaei F Seif M Gandomkar A Fattahi MR Hasanzadeh J. Agreement between laboratory-based and non-laboratory-based Framingham risk score in Southern Iran. Sci Rep. (2021) 11(1):11124. 10.1038/s41598-021-90188-5 34031448 PMC8144380

[34] Javed Z Kundi H Chang R Titus A Arshad H. Polysocial risk scores: implications for cardiovascular disease risk assessment and management. Curr Atheroscler Rep. (2023) 25(12):1059–68. 10.1007/s11883-023-01173-4 38048008

[35] Bazo-Alvarez JC Quispe R Peralta F Poterico JA Valle GA Burroughs M Agreement between cardiovascular disease risk scores in resource-limited settings: evidence from 5 Peruvian sites. Crit Pathw Cardiol. (2015) 14(2):74–80. 10.1097/HPC.0000000000000045 26102017 PMC4479423

[36] Muñoz VOM Ruiz Morales ÁJ Mariño Correa A Bustos CMM. Concordancia entre los modelos de SCORE y framingham y las ecuaciones AHA/ACC como evaluadores de riesgo cardiovascular. Rev Colomb Cardiol. (2017) 24(2):110–6. 10.1016/j.rccar.2016.06.013

[37] Mancera-Rincón P Giral-Giraldo HE Rizo-Tello VZ Barrera-Garavito ÉC. Concordancia entre escalas framingham ATP III, SCORE y ACC/AHA 2013 en una cohorte de pacientes en un hospital de cuarto nivel en el año 2015. Acta Med Colomb. (2018) 43(4):192–9. 10.36104/amc.2018.1269

[38] Muñoz OM Rodríguez NI Ruiz Á Rondón M. Validación de los modelos de predicción de framingham y PROCAM como estimadores del riesgo cardiovascular en una población colombiana. Rev Colomb Cardiol. (2014) 21(4):202–12. 10.1016/j.rccar.2014.02.001

[39] Lopez-Lopez JP Garcia-Pena AA Martinez-Bello D Gonzalez AM Perez-Mayorga M Muñoz Velandia OM External validation and comparison of six cardiovascular risk prediction models in the prospective urban rural epidemiology (PURE)-Colombia study. Eur J Prev Cardiol. (2025) 32(7):564–72. 10.1093/eurjpc/zwae242 39041366 PMC12066169

[40] World Health Organization. WHO global atlas of traditional, complementary and alternative medicine [Internet] (2005). Available online at: https://www.who.int/publications/i/item/9789241547178 (Accessed March 15, 2025).

[41] Rosende A DiPette D Brettler J Rodríguez G Zuniga E Connell K HEARTS In the Americas appraisal checklist and clinical pathway for comprehensive hypertension management in primary care. Rev Panam Salud Publica. (2022) 46:125. 10.26633/RPSP.2022.125 PMC9440731 36071921

[42] Skjødt M Tully MA Tsai LT Gejl KD Ørtenblad N Jensen K Need to revise classification of physical activity intensity in older adults? The use of estimated METs, measured METs, and V˙O₂ reserve. J Gerontol A Biol Sci Med Sci. (2024) 79(8):1. 10.1093/gerona/glae120 PMC11215700 38703071

[43] Kim J Tanabe K Yokoyama N Zempo H Kuno S. Association between physical activity and metabolic syndrome in middle-aged Japanese: a cross-sectional study. BMC Public Health. (2011) 11(1):624. 10.1186/1471-2458-11-624 21819591 PMC3199599

[44] Jeong SW Kim SH Kang SH Kim HJ Yoon CH Youn TJ Mortality reduction with physical activity in patients with and without cardiovascular disease. Eur Heart J. (2019) 40(43):3547–55. 10.1093/eurheartj/ehz564 31504416 PMC6855138

[45] Gerovasili V Agaku I Vardavas C Filippidis F. Levels of physical activity among adults 18–64 years old in 28 European countries. Prev Med. (2015) 81:87–91. 10.1016/j.ypmed.2015.08.005 26299619

[46] Selvin E Coresh J Golden SH Brancati FL Folsom AR Steffes MW. Glycemic control and coronary heart disease risk in persons with and without diabetes: the atherosclerosis risk in communities study. Arch Intern Med. (2005) 165(16):1910–6. 10.1001/archinte.165.16.1910 16157837

[47] Davis WA Gregg EW Davis TME. Temporal trends in cardiovascular complications in people with or without type 2 diabetes: the Fremantle diabetes study. J Clin Endocrinol Metab. (2020) 105(7):e2471–82. 10.1210/clinem/dgaa215 32352534

[48] Cai X Zhang Y Li M Wu JH Mai L Li J Association between prediabetes and risk of all-cause mortality and cardiovascular disease: updated meta-analysis. Br Med J. (2020) 370:m2297. 10.1136/bmj.m2297 32669282 PMC7362233

[49] Welsh C Welsh P Celis-Morales CA Mark PB Mackay D Ghouri N Glycated hemoglobin, prediabetes, and the links to cardiovascular disease: data from UK biobank. Diabetes Care. (2020) 43(2):440–5. 10.2337/dc19-1683 31852727

[50] Ross R Neeland IJ Yamashita S Shai I Seidell J Magni P Waist circumference as a vital sign in clinical practice: a consensus statement from the IAS and ICCR working group on visceral obesity. Nat Rev Endocrinol. (2020) 16(3):177–89. 10.1038/s41574-019-0310-7 32020062 PMC7027970

