# Supplementary material for: Comparative evaluation of ASCVD, SCORE-2, and HEARTS risk scores in Colombian adults at risk of type 2 diabetes: stratification, concordance, and associated factors
Source: Front Cardiovasc Med. 2026 Feb 4;12:1734611. doi: 10.3389/fcvm.2025.1734611 (PMC12913481; doi:10.3389/fcvm.2025.1734611)
Supplement: Supplementary file 1 [file Table1.docx]

***Supplementary Material***

| **Supplementary Material.** Cardiovascular risk according to ASCVD, SCORE-2 and the HEARTS risk scores. | | | |
| --- | --- | --- | --- |
| **Scale** | **CVR outcome/ duration** | **Risk classification** | **Total 868(100%)** |
| **ASCVD** | Incidence / 10 years | High (≥20%) | 31 (3.57) |
|  |  | Moderate (5%–<20%) | 348 (40.09) |
|  |  | Low (<5%) | 489 (56.34) |
| **SCORE-2** | Incidence and mortality / 10 years | High (≥5%) | 303 (34.91) |
|  |  | Moderate (≥1%–<5%) | 520 (59.91) |
|  |  | Low (<1%) | 45 (5.18) |
| **HEARTS** | Incidence of myocardial infarction, stroke, and mortality / 10 years | High (≥10%) | 41 (4.72) |
|  |  | Moderate (5%–10%) | 152 (17.51) |
|  |  | Low (<5%) | 675 (77.76) |
| Weighted Cohen´s Kappa = 0.10 (IC 95%: 0.08 - 0.13) para ASCVD vs. SCORE, 0.49 (IC 95%: 0.44 - 0.54) para ASCVD vs. HEARTS y 0.08 (IC 95%: 0.06 - 0.11) para-SCORE vs. HEARTS. | | | |
